# Seizures Causing Simultaneous Bilateral Neck of Femur Fractures

**DOI:** 10.1155/2019/4570578

**Published:** 2019-02-27

**Authors:** Dane Gary Maimin, Livan Meneses-Turino

**Affiliations:** ^1^Madadeni Hospital, Newcastle, South Africa; ^2^HOD Orthopaedics Northdale Hospital, Pietermaritzburg, South Africa

## Abstract

Neck of femur fractures are a ubiquitous injury seen by orthopaedic surgeons. In the elderly, these fractures usually occur after a low energy fall, and are invariably unilateral injuries. Bilateral neck of femur fractures have been reported in patients with metabolic bone disease, after electroconvulsive therapy, and in association with stress fractures. The otherwise healthy index patient in this case report presented most unusually, with simultaneous, bilateral neck of femur fractures that were sustained during a seizure.

## 1. Introduction

Hip fractures are a common occurrence throughout the world, with the United States alone seeing over 340,000 hip fractures per year [1] with incidences increasing dramatically with age, as bone density decreases and likelihood of falls increases. Between ages 60 and 85 years, the risk of fracture doubles for every 5- to 6-year increase in age [2]. The mechanism of injury is more commonly low energy trauma as this age group has higher rates of osteoporosis as well as osteomalacia, myeloma, renal osteodystrophy, and other metabolic and neoplastic bone diseases. The incidence of neck of femur fractures is highest in European caucasian populations. Denmark, Sweden, and Norway have incidences of 574, 563, and 539 per 100,000, respectively [3]. There is limited data regarding the incidence in South Africa and other African populations; however, several studies concur that the incidence in black African populations is among the lowest in the world [3, 4].

The vast majority of hip fractures occur unilaterally. Bilateral injuries occur very rarely but have been reported in patients with metabolic diseases, after electroconvulsive therapy, and due to stress fractures [4]. This report is interesting and uncommon as the patient presented with simultaneous, bilateral hip fractures sustained during a generalised tonic clonic seizure.

## 2. Case Report

The patient is a 69-year-old gentleman, who is HIV positive. His viral load is undetectable and his CD4 count is 417 cells/mm^3^. For six years he has been on first line antiretroviral therapy in compliance with the South African Department of Health guidelines i.e. a fixed-dose combination pill taken daily, containing tenofovir, emtricitabine, and efavirenz [5]. He has no history of seizures or other comorbidities. He is an independent and active member of the community. He does not smoke or abuse alcohol.

He first presented to his local clinic with a history of seizure-like activity beginning approximately one week prior. Subsequent to the onset of seizures, the patient had been unable to walk and was experiencing pain in both his hips. There was no history of trauma otherwise. At his clinic, he had a witnessed generalised tonic-clonic seizure which was aborted with a benzodiazepine. He was then referred to Madadeni Provincial Hospital.

**Figure 1:**
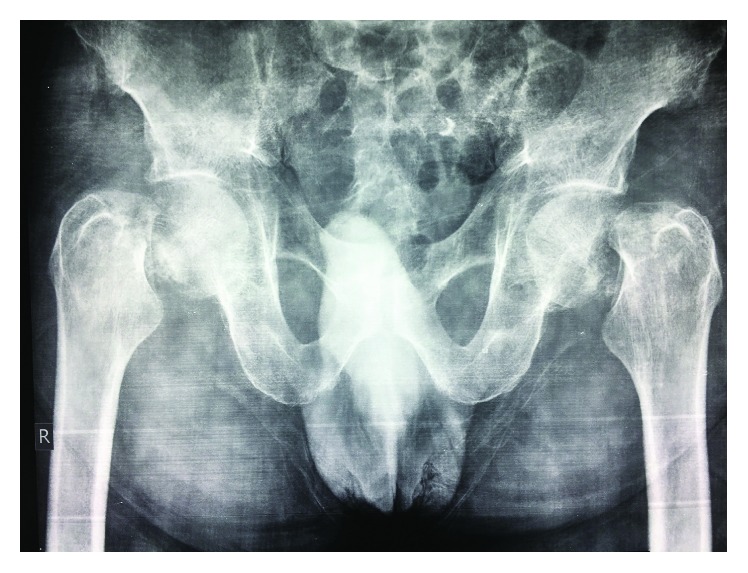


On presentation, the patient was noted to be drowsy but rousable, and cooperative. His vitals were normal. Orthopaedic examination showed bilaterally externally rotated lower limbs, with tenderness in the groin and pain on movement of his hips. He had no neurological deficit with a normal vascular exam. There were no signs of trauma or open wounds. Pelvic radiographs showed bilateral Garden 4 subcapital neck of femur fractures (Figure 1).

He was admitted for comanagement by internal medicine and orthopaedics. His medical workup revealed an electrolyte abnormality of severe hyponatraemia (115 mmol/L), and, as his other blood tests were normal and CT brain showing only age-related atrophy, the hyponatremia was attributed as the cause of the seizures. The hyponatremia was corrected and he had no further seizures in the ward.

**Figure 2:**
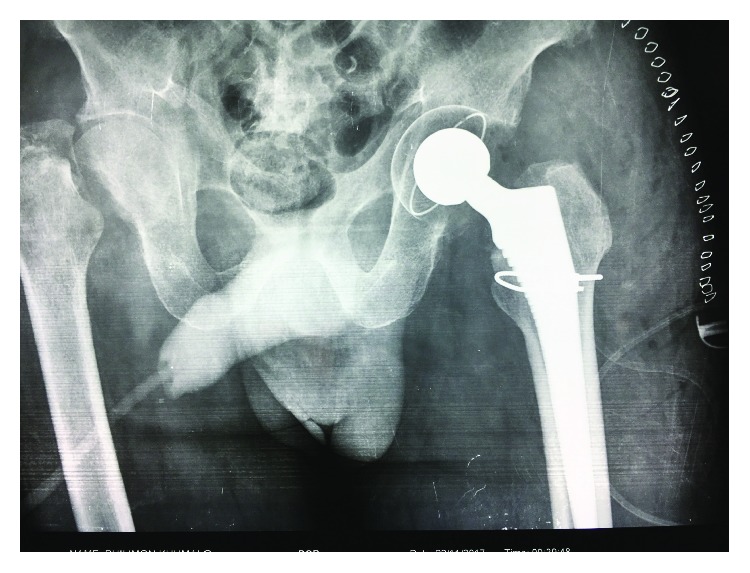


**Figure 3:**
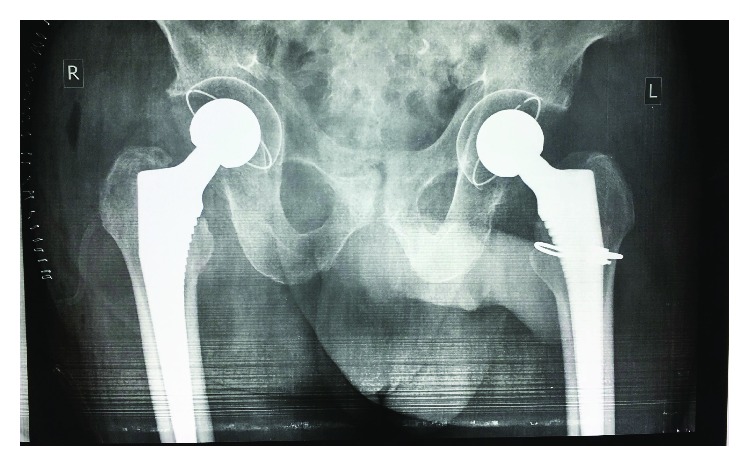


He was kept in bilateral skin traction during his medical optimisation. Staged, bilateral total hip arthroplasties was ultimately performed. Due to lack of theatre time and a high patient load at Madadeni Hospital, the patient waited almost two weeks for his first surgery (Figure 2) and another month before his second surgery (Figure 3). The patient was assessed as a Dorr B, had good intraoperative bone quality and both arthroplasties were thus uncemented. During the first (left) arthroplasty, there were concerns of fracture extension into the intertrochanteric region, and a cabling system was used to reinforce the stem insertion. For both surgeries, a Mathys RM Pressfit cup, with symarec head and a Zimmer Avenir stem, was used. Several bone biopsies were also taken during the first surgery to exclude a pathological fracture. All biopsies were normal.

Postoperatively, the patient was kept on DVT prophylaxis and began in-bed hip and knee range of motion exercises on day two after surgery. The patient was mobilising with the assistance of a physiotherapist and a walking frame within a week of his second surgery and was discharged home with no postoperative complications.

## 3. Discussion

The strong muscle contractions during seizures are known to cause fractures and/or dislocations with an incidence of 1.1% following a convulsion [6]. However, there were very few reported cases of bilateral hip fractures sustained during seizure [4, 6–11]. Those that do occur tend to have an underlying metabolic bone disease such as osteomalacia or decreased bone density as a result of chronic antiepileptic treatment.

Patients presenting with seizures may have several potential reasons for a decreased level of consciousness and further more may need to be sedated and or ventilated as part of their treatment. As such, musculoskeletal injuries, including neck of femur fractures which have very subtle clinical signs, may initially be missed [7]. Any patient presenting with seizures requires a thorough musculoskeletal examination to prevent complications such as avascular necrosis, osteoarthritis, nonunion, functional disability, and legal consequences [10]. This is especially relevant in intracapsular neck of femur fractures in younger patients.

## 4. Conclusion

Musculoskeletal complications postseizures are rare but easily missed. All physicians treating patients with seizures should maintain a high index of suspicion to avoid missing injuries and incurring the complications that may follow.

## References

[1] Brauer C. A., Coca-Perraillon M., Cutler D. M., Rosen A. B. (2009). Incidence and mortality of hip fractures in the United States. *Journal of the American Medical Association*.

[2] Kim S. H., Meehan J. P., Blumenfeld T., Szabo R. M. (2012). Hip fractures in the United States: 2008 nationwide emergency department sample. *Arthritis Care & Research*.

[3] Kanis J. A., Odén A., McCloskey E. V. (2012). A systematic review of hip fracture incidence and probability of fracture worldwide. *Osteoporosis International*.

[4] Paruk F., Matthews G., Cassim B. (2017). Osteoporotic hip fractures in black South Africans: a regional study. *Archives of Osteoporosis*.

[5] Meintjies G., Moorhouse M. A., Carmona S. (2017). Adult antiretroviral therapy guidelines 2017. *Southern African Journal of HIV Medicine*.

[6] Cagırmaz T., Yapici C., Orak M. M., Guler O. (2015). Bilateral femoral neck fractures after an epileptic attack: a case report. *International Journal of Surgery Case Reports.*.

[7] Shah H. M., Grover A., Gadi D., Sudarshan K. (2014). Bilateral neck femur fracture following a generalized seizure- a rare case report. *The Archives of Bone and Joint Surgery*.

[8] Joshy S. (2004). Bilateral femoral neck fracture following convulsions: pitfalls in early diagnosis and management. *The Internet Journal of Orthopedic Surgery*.

[9] Powell H. D. W. (1960). Simultaneous bilateral fractures of the neck of the femur. *The Journal of Bone and Joint Surgery. British volume*.

[10] Grimaldi M., Vouaillat H., Tonetti J., Merloz P. (2009). Simultaneous bilateral femoral neck fractures secondary to epileptic seizures: treatment by bilateral total hip arthroplasty. *Orthopaedics & Traumatology, Surgery & Research*.

[11] Ribacoba-Montero R., Salas-Puig J. (1997). Simultaneous bilateral fractures of the hip following a grand mal seizure. An unusual complication. *Seizure*.

